# Pd-Decorated SnO_2_ Nanofilm Integrated on Silicon Nanowires for Enhanced Hydrogen Sensing

**DOI:** 10.3390/s25030655

**Published:** 2025-01-23

**Authors:** Tiejun Fang, Tianyang Mo, Xianwu Xu, Hongwei Tao, Hongbo Wang, Bingjun Yu, Zhi-Jun Zhao

**Affiliations:** 1 Tribology Research Institute, School of Mechanical Engineering, Southwest Jiaotong University, Chengdu 610031, China; chen11_21@163.com (T.F.); 15194879258@163.com (T.M.); xxw51swjtu@163.com (X.X.); bingjun@swjtu.edu.cn (B.Y.); 2 Institute of Smart City and Intelligent Transportation, Southwest Jiaotong University, Chengdu 611756, China; hwtao@swjtu.edu.cn (H.T.); hongbowang@swjtu.edu.cn (H.W.)

**Keywords:** hydrogen sensor, silicon nanowire, MaCE, Pd-decorated SnO_2_ nanofilm, response

## Abstract

The development of reliable, highly sensitive hydrogen sensors is crucial for the safe implementation of hydrogen-based energy systems. This paper proposes a novel way to enhance the performance of hydrogen sensors through integrating Pd-SnO_2_ nanofilms on the substrate with silicon nanowires (SiNWs). The samples were fabricated via a simple and cost-effective process, mainly consisting of metal-assisted chemical etching (MaCE) and electron beam evaporation. Structural and morphological characterizations were conducted using scanning electron microscopy (SEM) and X-ray photoelectron spectroscopy (XPS). The experimental results showed that, compared to those without SiNW structure or decorative Pd nanoparticles, the Pd-decorated SnO_2_ nanofilm integrated on the SiNW substrates exhibited significantly improved hydrogen sensing performance, achieving a response time of 9 s at 300 °C to 1.5% H_2_ and a detection limit of 1 ppm. The enhanced performance can be primarily attributed to the large surface area provided by SiNWs, the efficient hydrogen spillover effect facilitated by Pd nanoparticles, and the abundant oxygen vacancies present on the surface of the SnO_2_ nanofilm, as well as the Schottky barrier formed at the heterojunction interface between Pd and SnO_2_. This study demonstrates a promising approach for developing high-performance H_2_ sensors characterized by ultrafast response times and ultralow detection limits.

## 1. Introduction

As global initiatives to advance renewable energy intensify, hydrogen is being increasingly recognized as a clean and efficient energy carrier. Its widespread applications in fuel cells, portable energy storage, and the hydrogen industry are driving the development of advanced hydrogen safety monitoring technologies [1,2]. However, the flammability of hydrogen, combined with its high diffusivity in air, significantly increases the risk of leaks. Consequently, detecting hydrogen concentration and potential leaks has become a critical measure for ensuring both safety and property protection [3,4]. Additionally, various industrial sectors, including metallurgy, petrochemicals, automotive exhausts, and nuclear energy, require hydrogen sensors that can operate effectively in high-temperature and challenging environments. For example, monitoring hydrogen levels in molten aluminum during the casting process [5] and ensuring the safety of nuclear reactors at power plants [6] highlight the need for robust hydrogen sensing solutions. Current hydrogen detection techniques encompass a variety of methods, including electrochemical sensors, resistive sensors, catalytic sensors, and optical sensors [7,8,9,10]. Among these, metal oxide semiconductor (MOS) gas sensors are particularly promising for hydrogen detection due to their low cost, excellent sensing performance, reliability, and facilitated fabrication [11]. Fundamentally, MOS-based gas sensors operate on the principle that the electrical signal (either current or resistance) varies in response to the interaction between the target gas and the oxygen adsorbed on the surface of the MOS [12]. Among various semiconducting metal oxides, SnO_2_, as a n-type semiconductor, has become a leading choice in gas sensor research, owing to its exceptional physical and chemical properties, as well as its superior gas sensing capabilities [13,14]. However, bare SnO_2_ has challenges in meeting the demands for developing high-performance hydrogen sensors [15,16]. Consequently, to enhance the sensing performance of SnO_2_ gas sensors, many researchers are pursuing an effective method of doping with noble metals (e.g., Ag, Au, Pt) [17,18,19]. Notably, palladium (Pd), as a typical catalyst, can significantly accelerate the adsorption and reaction of hydrogen molecules, thereby enhancing the selectivity and sensitivity of hydrogen sensors. For example, Li et al. fabricated ultrathin nanosheet hydrogen gas sensors based on SnO_2_, demonstrating that the 1.0 wt% Pd/SnO_2_ sensor exhibited superior sensitivity, achieving a lower detection limit and rapid response/recovery time of 21 s and 13 s towards 20 ppm H_2_ at 220 °C, respectively. Furthermore, it showed a marked improvement in selectivity for H₂ compared to pure SnO_2_ sensors at 300 °C [20]. Meng et al. reported that Pd/SnO_2_ nanoparticles outperformed pure SnO₂ in hydrogen sensing, achieving a response magnitude of 254 and rapid response/recovery time of 1 s/22 s, respectively, in response to 500 ppm H_2_ at 125 °C [21]. Meanwhile, Kim et al. fabricated both bare and Pd-decorated SnO_2_ nanowires using a vapor–liquid–solid method, revealing that the Pd/SnO_2_ nanowire sensor increased its response at 1 ppm H₂ from 6.88 to 16.95 at 300 °C [22]. Additionally, the structural properties of gas sensors play a crucial role in the advancement of sensing systems with enhanced characteristics [23,24]. Various nanostructures including nanotubes, nanorods, and nanowires, have been extensively utilized in gas sensors due to their excellent gas sensing performance [25,26,27]. Among these, SiNWs have attracted significant attention in recent years due to their unique chemical properties and potential applications in nano/optoelectronics, solar cells, and chemo/biosensors [28,29,30]. To fabricate SiNWs, various methods have been developed, including vapor–liquid–solid synthesis (VLS), reactive-ion etching (RIE), and metal-assisted chemical etching (MaCE) [31,32,33]. Particularly, MaCE is notable for its simplicity, cost-effectiveness, and flexibility in producing vertical SiNWs at the wafer scale, allowing for controlled morphology and microstructure [34]. In the realm of gas sensing applications, the SiNW structure emerges as a particularly attractive candidate due to its large specific surface area, high chemical activity, and excellent compatibility for doping with other sensing materials. For example, Cuscunà et al. successfully fabricated a chemo-resistive sensor based on SiNWs, achieving a NO_2_ detection limit down to a few parts per billion at room temperature [35]. Similarly, Qin et al. demonstrated that SiNW/WO_3_ nanowires exhibited good selectivity to NO_2_ and an ultrafast response time of less than 1 s to 0.5–5 ppm NO_2_ at room temperature [36]. Furthermore, Noh et al. reported that Pd-coated rough silicon nanowires displayed a rapid response time (<3 s) and low detection limit (~5 ppm) at room temperature [37]. Although both the SiNW structure and Pd-decorated SnO_2_ materials have been individually and extensively studied to enhance gas sensing performance, their integration within hydrogen sensors remains a largely unexplored area.

Therefore, in this work, we fabricated the hydrogen sensor of Pd-decorated SnO_2_ sensing nanofilm integrated on SiNW substrate to study the potential gas sensing performance. The SiNW substrate was fabricated via the MaCE process with a silver holey mask, and the mask was previously prepared by annealing the deposited 30 nm-thick Ag nanofilm, which is a solid-state dewetting process selected to pattern the Ag mask instead of using lithograph techniques or other methods, because it is a simple and cost-effective approach. Afterwards, a 40 nm-thick SnO_2_ nanofilm and a 1 nm-thick Pd nanofilm were deposited on the SiNW substrates by electron beam evaporation, respectively. The synthesized hydrogen sensors based on Pd-decorated SnO_2_ nanofilm integrated on SiNW substrate showed a high response value greater than 9, a rapid respond to 1.5% H_2_ (a respond time of 9s), and a detection limit of 1 ppm H_2_. Such an excellent sensing performance could probably be attributed to the synergistic effect of SnO_2_ nanofilm, Pd nanoparticles, and the p-n heterojunction formed at the interface between SnO_2_ and SiNWs, as well as plenty of active sites provided by the SiNW structure with large surface area.

## 2. Experimental Section

### 2.1. Materials

Hydrofluoric acid (AR, 40%), nitric acid (AR, 65%), acetone (AR, 99%), ethanol (AR, 99%), and hydrogen peroxide (AR, 30%) were bought from Chengdu Kelong Chemical Co., Ltd. (Chengdu, China). SnO_2_ particles (1–3 mm, 99.99%), Ag particles (φ 2 mm ×5 mm, 99.99%), and Pd particles (φ 2 mm × 5 mm, 99.95%) to be used as target materials for electronic beam evaporation were bought from Beijing Dream Material Technology Co., Ltd. (Beijing, China). p-type silicon wafer (φ 50.8 mm, <100>, 1–10 Ω·cm, 280 ± 15 μm) were bought from Wuxi Zhonghuixin Technology Co., Ltd. (Wuxi, China).

### 2.2. Fabrication of Pd-Decorated SnO_2_ Nanofilm on SiNW Substrate

**Figure 1:**
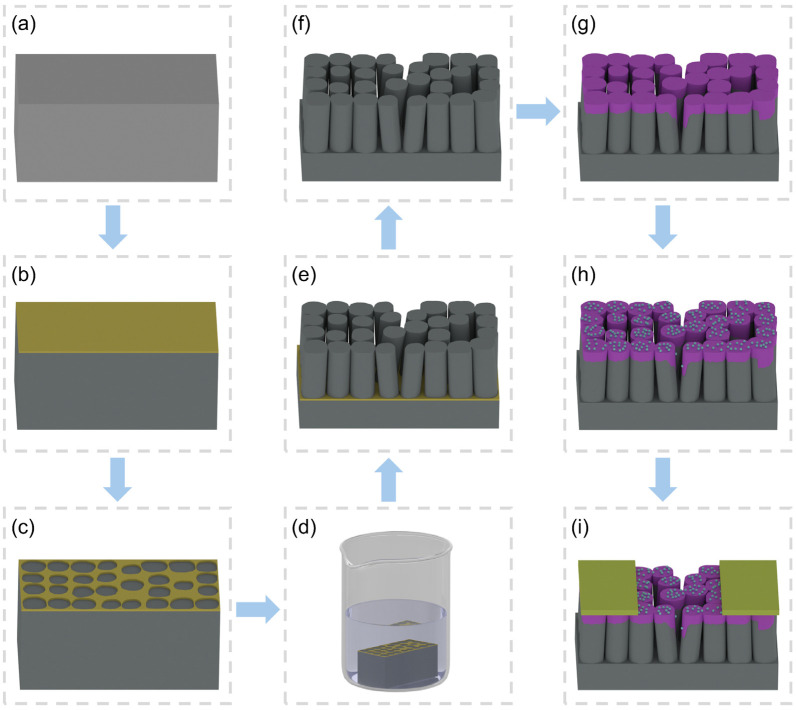
Schematic illustration showing the fabrication process of Pd-decorated SnO2 Nanofilm on SiNW substrate. (**a**) A rinsed p-Si slice, (**b**) 30 nm-thick silver film deposited by electron beam evaporation, (**c**) a nanostructured silver holey mask by annealing, (**d**) the sample under the process of MaCE, (**e**) SiNW substrate with Ag nanoparticles remained after MaCE, (**f**) SiNW substrate after removing Ag nanoparticles, (**g**) 40 nm-thick SnO_2_ film deposited by electron beam evaporation, (**h**) 1 nm-thick Pd film deposited by electron beam evaporation, and (**i**) Ag electrodes deposited by electron beam evaporation.

**Figure 2:**
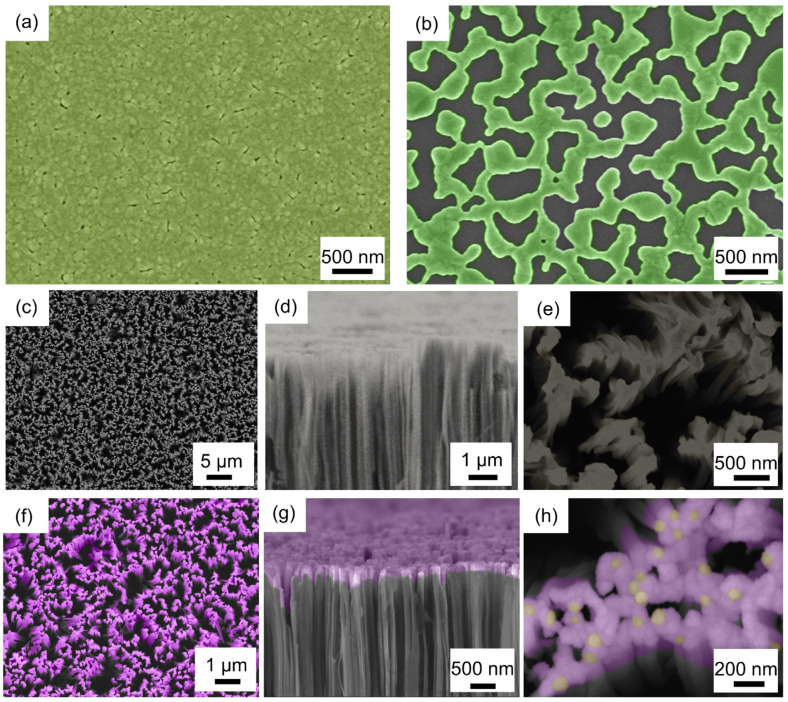
Top-view SEM images of 30 nm-thick Ag film (**a**), a silver holey mask (**b**), vertical SiNW structure after MaCE (**c**,**e**) and Pd (1 nm)-decorated SnO_2_ nanofilm (40 nm) on SiNWs (**f**,**h**). Cross-sectional SEM images of vertical SiNWs after MaCE (**d**) and Pd (1 nm)-decorated SnO_2_ nanofilm (40 nm)-covered SiNWs (**g**).

**Figure 3:**
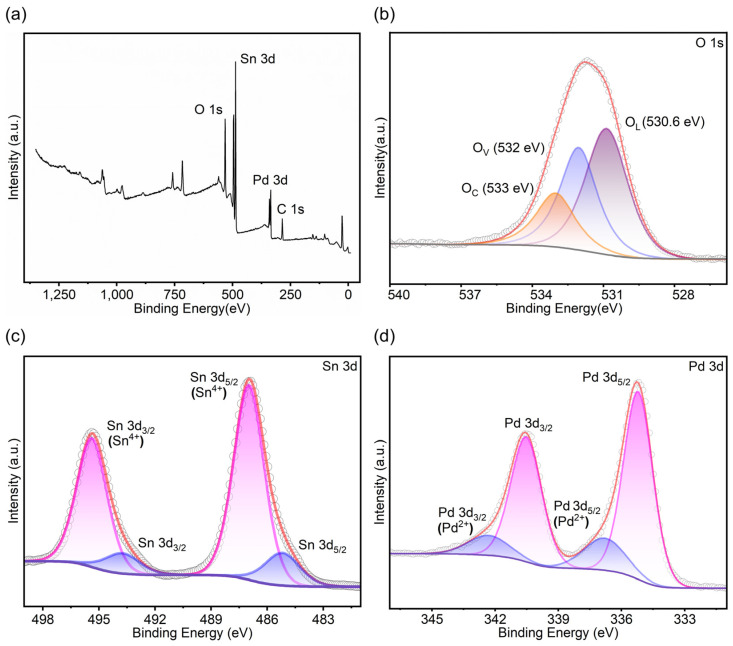
XPS spectra of Pd-decorated SnO_2_ nanofilm integrated on SiNWs: (**a**) survey spectrum, (**b**) O 1s spectrum, (**c**) Sn 3d spectrum, and (**d**) Pd 3d spectrum.

As illustrated in Figure 1, the fabrication process of the porous Pd-decorated SnO_2_ nanofilm can be divided into three main steps: (1) the preparation of a nanostructured silver holey mask (Figure 1a–c), (2) the fabrication of a vertical SiNW substrate (Figure 1d–f), and (3) the deposition of the Pd-decorated SnO_2_ nanofilm onto the SiNW substrate (Figure 1g–i).

#### 2.2.1. Preparation of a Nanostructured Silver Holey Mask

P-type silicon wafers were cut into 4 mm × 1 mm pieces, which were then ultrasonically cleaned in acetone and ethanol for 5 min, respectively (Figure 1a), followed by a rinse with deionized (DI) water. Subsequently, a 30 nm-thick silver nanofilm was deposited onto the cleaned silicon substrates using electron beam evaporation (Figure 1b). To conduct the solid-state dewetting of the silver nanofilm, the samples were put in a preheated muffle furnace at 250 °C and annealed for 30 min. Afterwards, the samples were taken out from the furnace and cooled down to room temperature under environmental circumstances. Then, a nanostructured silver holey mask was prepared (Figure 1c).

#### 2.2.2. Fabrication of a Vertical SiNW Substrate

The substrates covered with silver holey masks were immersed in MaCE solution consisting of 4.8 M HF and 0.05 M H_2_O_2_ for 3 h at room temperature (Figure 1d). In this step, the Ag nanofilm catalyzed H_2_O_2_ reduction, injecting holes through the Ag/Si Schottky barrier and oxidizing Si that dissolved in the presence of HF [38], and only the silicon in contact with Ag was etched (Figure 1e). Afterwards, the samples were put into a HNO_3_ solution (65 wt%) for 40 min to remove Ag nanoparticles, followed by rinsing with ethanol and DI water, respectively. Consequently, a vertical SiNW substrate was fabricated (Figure 1f).

#### 2.2.3. Deposition of Pd-Decorated SnO_2_ Nanofilm on SiNW Substrate

In this work, all the films were deposited using electron beam evaporation, which is a physical vapor deposition technique. Before the deposition, the chamber should be evacuated to a high vacuum with a pressure of 5 × 10^−4^ Pa, and the depositing rate should be set to 0.5 Å/s. First, a 40 nm-thick SnO_2_ nanofilm was deposited on each SiNW substrate (Figure 1g), followed by the deposition of a 1 nm-thick Pd nanofilm to decorate the SnO_2_ layer (Figure 1h). Subsequently, silver electrodes with an area of 1 mm × 1 mm were deposited on the samples, while the sensing zone measured 1 mm × 2 mm (Figure 1i). To better investigate SiNWs and different thicknesses of Pd films and SnO_2_ fims on the gas sensing performance, 7 different kinds of sensors were fabricated, and they are shown in detail in Table 1.

### 2.3. Characterization

The scanning electron microscopy (JSM 7800F Prime, JEOL Ltd., Tokyo, Japan) with energy-dispersive X-ray spectroscopy EDS was employed to explore the morphology and composition of the samples. X-ray photoelectron spectroscopy (XPS, Thermo Scientific ESCALAB 250Xi, Al K-Alpha X-ray, Thermo Fisher Scientific Ltd., Waltham, MA, USA) was employed to analyze the surface chemical composition and valence states of the elements. X-ray diffraction (XRD, Empyrean, PANalytical B.V.Ltd., Almelo, The Netherlands) with Cu-Kα radiation (λ = 1.54178 Å) was employed to study the crystallinity.

### 2.4. Hydrogen Gas Sensing Tests

The hydrogen gas sensing tests were conducted on an optoelectrical integrated test platform (CGS-MT, Beijing Sino Aggtech Co., Ltd., Beijing, China) under dynamic testing circumstances. As is shown in Figure S1, the platform consists of a gas distribution system, a temperature control system (ranging from 25 °C to 400 °C), and a gas sensing analysis system. The gas distribution system was devised to regulate the concentration of the target gas by blending it with a balance gas in specific ratios and to automatically enable the switching between the target gas and the background gas. The temperature control system was accountable for providing a stable operating temperature during gas sensing tests. Meanwhile, the gas sensing analysis system measured variations in the electrical performance of the sensor and sent the relevant signals to the computer, which would then display these signals on the screen in the form of current, voltage, or resistance. The response of the sensor is defined as S = R_a_/R_g_, and R_a_ is the stable resistance in air atmosphere, while R_g_ is the stable resistance in the target gas. The response time and recovery time are defined as the period required for 90% resistance change during H_2_ adsorption and desorption, respectively.

## 3. Results and Discussion

### 3.1. Morphology and Material

Figure 2a shows the SEM image of an even silver nanofilm with a thickness of 30 nm, which was deposited by electron beam evaporation. The silver mask with high-density holes, which has a direct impact on the formation of the vertical SiNWs, is shown in Figure 2b. Prior to this, to determine the appropriate parameters for the formation of the silver holey masks, the silver nanofilms with thicknesses of 10 nm, 20 nm, 25 nm, 30 nm, 35 nm, and 40 nm on silicon substrates were fabricated and annealed at 250 °C for 30 min. The results indicated that the 30 nm-thick silver nanofilm had a good hole nanostructure (Figure S2d), while the 10 nm-, 20 nm-, and 25 nm-thick silver nanofilms became separate Ag particles (Figure S2a–c), the 35 nm- and 40 nm-thick silver nanofilms remained almost as an integrated nanofilm with few and small holes (Figure S2e,f). Figure S3 shows that 250 °C could be an appropriate temperature value based on the temperature tests. Because the 30 nm-thick silver nanofilm annealed at 250 °C for 30 min turned out to have a nanostructure with high-density holes (Figure S3b), while for the 30 nm-thick silver nanofilms annealed at 180 °C and 250 °C for 30 min, respectively, they either presented a nanofilm with small holes (Figure S3a) or presented separate Ag particles (Figure S3c), which would adversely affect the subsequent MaCE process.

Figure 2c–e displays the structures of the vertical SiNWs after MaCE. Most of the nanowires are upright, and the average height of the nanowires is about 20–30 nm. The nanowires are densely distributed (shown in Figure 2c), due to which the upcoming deposited nanofilm would have a high surface area for gas sensing. Figure 2f–h clearly shows the structures of the Pd (1 nm)-decorated SnO_2_ nanofilm (40 nm) integrated on SiNWs. As shown in Figure 2g,h, it is noteworthy that the vertical SiNWs are successfully coated, and parts of them are connected on the upper by the deposited SnO_2_ nanofilm, which is decorated by Pd nanoparticles. It might offer conditions for the current to flow along the Pd-decorated SnO_2_ nanofilm deposited on the vertical SiNWs. To better confirm the distribution of the SnO_2_ nanofilm on the SiNW substrate, a 130 nm-thick SnO_2_ film was deposited, and the SEM images in Figure S4 show that SnO_2_ nanofilms are primarily distributed on the upper surface of SiNWs.

We utilized XPS spectra to analyze the composition and the specific chemical states of the silicon nanowire-based SnO_2_ nanofilm decorated by Pd nanoparticles. In Figure 3a, the XPS survey spectra of the Pd-decorated SnO_2_ nanofilm reveal the existence of O, Sn, Pd, and C elements, and C may result from inadvertent carbon contamination. As shown in Figure 3b, the O 1s signal splits into three distinct peaks at 533 eV, 532 eV, and 530.6 eV. Lattice oxygen (O_L_) has no influence in gas detection due to its stable chemical structure. In contrast, the oxygen vacancy (O_V_) and chemisorbed oxygen species (O_C_) are the key factors that enhance the gas sensing performance of MOS-based sensors [39]. Figure 3c displays two Sn 3d peaks split into Sn 3d_5/2_ (Sn^4+^), Sn 3d_3/2_ (Sn^4+^), Sn 3d_5/2_ (Sn), and Sn 3d_3/2_ (Sn) peaks, confirming the presence of both SnO_2_ and Sn in the Pd-decorated SnO_2_ nanofilm on SiNWs. According to the area size of the related peaks, it can tell that SnO_2_ accounts for a much larger proportion than Sn element substance [40]. The XPS spectrum of the Pd 3d in Figure 3d exhibits two well-defined peaks of Pd 3d_5/2_ (335.3 eV) and Pd 3d_3/2_ (340.5 eV), which further splits into four distinct peaks through fitting analysis. The XPS spectrum with the two peaks at 336.7 eV and 342.1 eV is associated with Pd^0^ [41], while the XPS spectrum with the other two peaks at 335.2 eV and 340.5 eV can be attributed to Pd^2+^. The XPS spectra suggest the presence of both Pd and PdO in the Pd-decorated SnO_2_ nanofilm. Judging from the area size of the relevant peaks, it can be inferred that the proportion of Pd is significantly higher than that of PdO [42].

**Figure 4:**
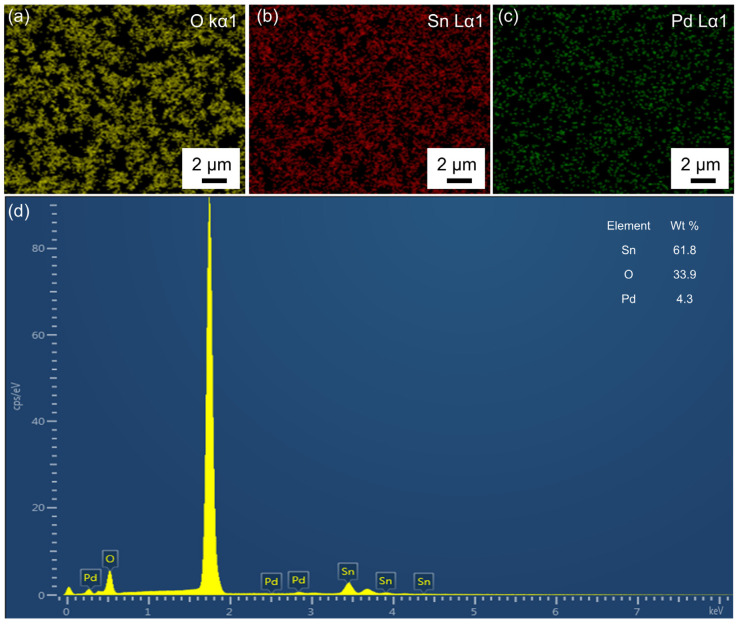
EDS images including the element content of O (**a**), Sn (**b**), Pd (**c**), and EDS spectra (**d**) of Pd-decorated SnO_2_ nanofilm on SiNWs.

The EDS spectrum in Figure 4d demonstrates the element proportions of O, Sn, and Pd to be 33.9 wt%, 61.8 wt% and 4.3 wt%, respectively, which confirms the existence of the three elements (O, Sn, and Pd) in the nanofilm. The EDS element images of the Pd-decorated SnO_2_ nanofilm on SiNWs are displayed in Figure 4a–c. Clearly, O, Sn, and Pd elements are present in the nanofilm and are distributed across the upper surface of the SiNWs. Therefore, the EDS spectrum and elemental mapping analysis collectively demonstrate the successful deposition of the Pd-decorated SnO_2_ nanofilm on the SiNW substrate.

### 3.2. Gas Sensing Properties

**Figure 5:**
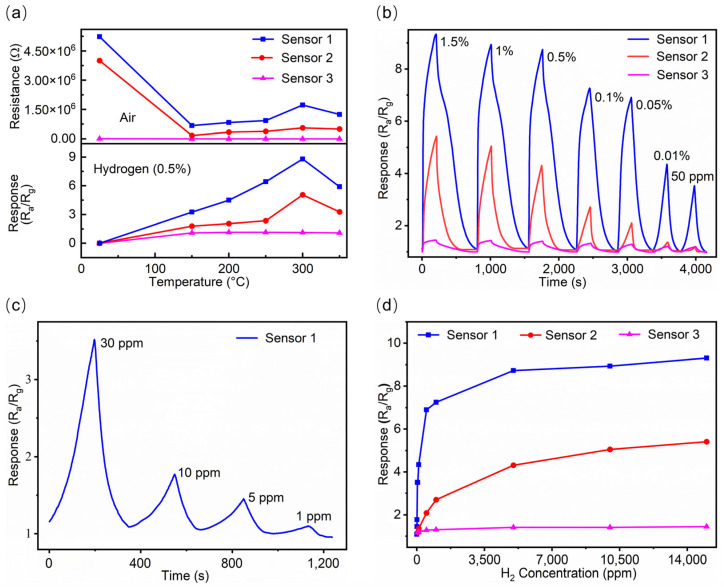
(**a**) The baseline resistance curves of the sensors to air at various operating temperatures (**upper**), and the response curves of the sensors to 0.5% H_2_ at various operating temperatures (**below**). (**b**) The response curves of the sensors to the high ppm level H_2_ concentrations at 300 °C. (**c**) The response curves of sensor 1 to the low ppm level H_2_ concentrations at 300 °C. (**d**) The response curves of the three sensors to different H_2_ concentrations (1–15,000 ppm) at 300 °C.

The upper graph of Figure 5a shows the baseline resistance changes in all sensors in the air at various temperatures. The sensors with a SiNW structure (Sensor 1 and 2) exhibited higher resistance than the sensor with a non-SiNW structure (Sensor 3). The increased baseline resistance is likely due to the silicon nanowire structure with a large specific surface area. Additionally, Sensor 1 displays higher baseline resistance than the other two, which may be caused by the decorative Pd particles. The upper graph also illustrates the trend of resistance changes with temperature. As the temperature rises from room temperature, resistance decreases significantly due to the movement of energized electrons into the conduction band [43]. However, with further increases in temperature, the baseline resistance rises, because more activated oxygen ions capture electrons from the conduction band, leading to an increase in resistance [44]. The lower graph in Figure 5a depicts the response of SnO_2_ nanofilm-based sensors to 0.5% H_2_ at different temperatures spanning from 25 °C to 350 °C. The findings indicate that the best operating temperature for all three SnO_2_-based sensors could be 300 °C, at which the sensors exhibit the maximum response. Many reports have demonstrated that the response performance of MOS-type gas sensors is greatly affected by the working temperature, which influences the absorption and diffusion capabilities of the relevant gases. At low temperatures, limited thermal energy would result in weak redox reactions and poor responses, while the desorption activity is faster than the adsorption, reducing gas absorption and response at elevated temperatures. Thus, the best working temperature is where gas absorption and chemical activating energy become balanced. In this work, the testing data show that the perfect operating temperature of all the SnO_2_-based sensors would be 300 °C, at which the sensors display the best response.

Figure 5b shows the gradient response of three sensors to the high ppm level H_2_ concentrations spanning from 50 ppm to 15,000 ppm at 300 °C. Sensor 1, which has both a SiNW structure and decorative Pd particles, consistently outperforms Sensor 2 (only with SiNW) across all concentrations. Sensor 2, in turn, has a higher response than Sensor 3, which lacks both features. Notably, at 50 ppm H_2_, Sensor 1 maintains a response of 3.5, while Sensors 2 and 3 reach their detection limits with a response value around 1. Furthermore, Figure 5c shows that Sensor 1 exhibits a strong response to the low ppm level H_2_ concentrations (1 ppm to 30 ppm) at 300 °C, maintaining a response value of 1.2 at 1 ppm H_2_. Figure 5d illustrates the response of each sensor at varying gas concentrations at 300 °C, revealing that the response intensifies with increasing concentration. Besides that, it also shows that Sensor 1 exhibits a faster response increase compared to the other two sensors. All these enhanced performances of Sensor 1 may be ascribed to the synergistic effect of silicon nanowires, SnO_2_ nanofilm, and Pd nanoparticles.

**Figure 6:**
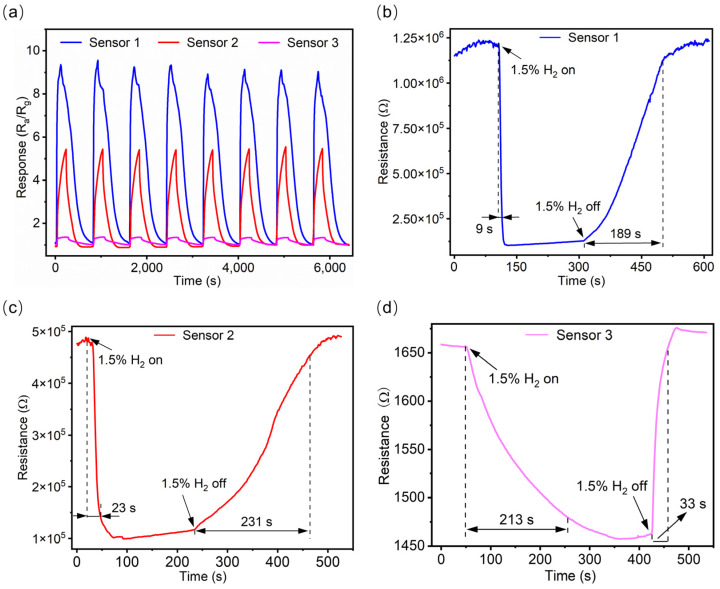
(**a**) The cycling response curves of the sensors to 1.5% H_2_ at 300 °C. The response and recovery time of Sensor 1 (**b**), Sensor 2 (**c**), and Sensor 3 (**d**) to 1.5% H_2_ at 300 °C.

Figure S5 shows that the response times and respond values of Sensors 4 and 5 towards 1.5% H_2_ at 300 °C are 123 s, 199 s, 1.7, and 1.9, respectively. In contrast, Sensor 2 exhibits a response time of 23 s (Figure 6c) and a response greater than 5 (Figure 6a) under the same conditions. This suggests that a 40 nm-thick SnO_2_ film offers better hydrogen sensing performance compared to 30 nm- and 50 nm-thick SnO_2_ films when SiNWs are used as the substrate in this work. Similarly, Figure S6 shows that the response times and response values of Sensors 6 and 7 towards 1.5% H_2_ at 300 °C are 57 s, 142 s, 2.2, and 1.6, respectively. In comparison, Sensor 1 has a response time of 9 s (Figure 6b) and a response greater than 9 (Figure 6a) under the same working conditions. This indicates that a 1 nm-thick Pd film decorating a SiNW-based 40 nm-thick SnO_2_ film provides superior hydrogen sensing performance compared to the same SnO_2_ films decorated with 3 nm- and 6 nm-thick Pd films.

**Figure 7:**
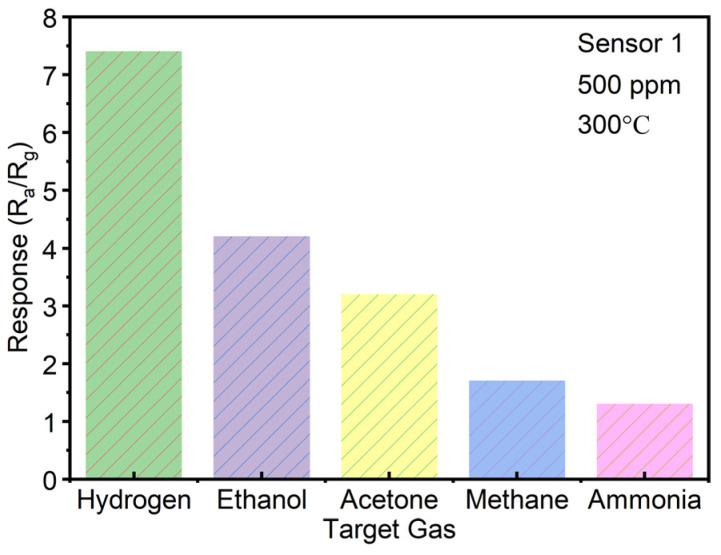
Selectivity test histogram of Sensor 1 towards 500 ppm of different gases at 300 °C.

**Figure 8:**
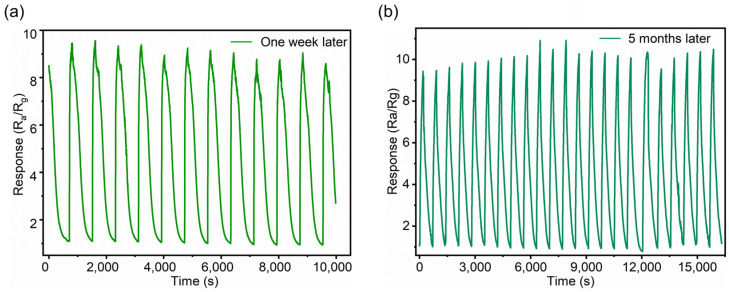
Long-term stability test graphs of Sensor 1 towards 1.5% H_2_ at 300 °C. (**a**) The test after one week; (**b**) the test after 5 months.

Additionally, for the repeatability tests of all three sensors in response to 1.5% H_2_ gas (15,000 ppm), eight cycles were conducted at 300 °C. The results, presented in Figure 6a, demonstrate that all three sensors exhibit good repeatability in gas sensing. Notably, due to the enhancement provided by the SiNW structure and the decorative Pd particles, Sensor 1 achieves a response value greater than 9, significantly surpassing the response values of Sensor 2 and 3. Figure 6b–d presents the response and recovery times of Sensor 1, Sensor 2, and Sensor 3 towards 1.5% H_2_ at 300 °C. All sensors demonstrate superior response performance to H_2_ compared to their recovery phase. Particularly, Sensor 1 demonstrates the faster respond speed than Sensors 2 and 3, with a response time reduced to 9 s. This rapid response may be triggered collaboratively by the SiNW structure, SnO_2_ nanofilm, and the decorative Pd particles. Table S1 compares the H_2_ gas detection limits of several SnO_2_ sensors containing Pd with the present sensor. The current sensor shows a lower detection limit than the other reported sensors, indicating its superior performance in detection of low concentrations of H_2_. In Figure 7, Sensor 1 exhibits a markedly stronger response to 500 ppm H_2_ at 300 °C compared to other target gases, including ethanol, acetone, methane, and ammonia. The results indicate that Sensor 1 demonstrates good selectivity for H_2_. In Figure 8a, Sensor 1 maintains a consistent response of approximately 9 to 1.5% H_2_ at 300 °C over 10 cycles after one week. Remarkably, as shown in Figure 8b, even after 5 months, Sensor 1 continues to show stable performance, with a response of around 9 to 1.5% H_2_ at 300 °C over 20 cycles. These findings suggest that Sensor 1 exhibits excellent stability.

### 3.3. Hydrogen Sensing Mechanism

**Figure 9:**
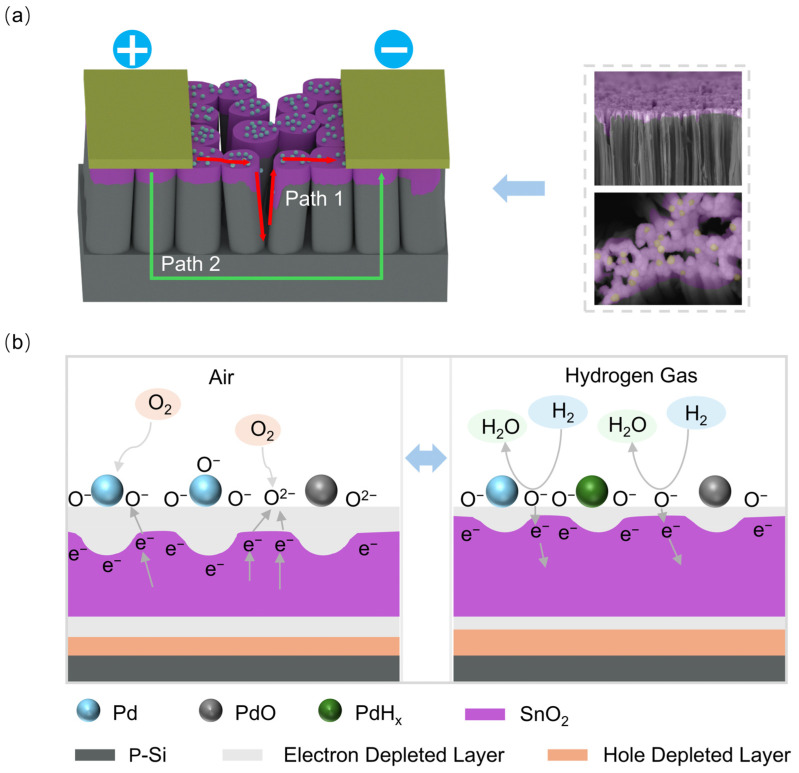
(**a**) The overall structure and electrical circuit; (**b**) gas sensing mechanism of Sensor 1.

In this part, we will investigate the sensing mechanisms of the hydrogen sensors of Pd-decorated SnO_2_ nanofilms integrated on SiNWs (Sensor 1) through a comparative analysis with Sensors 2 and 3. Outwardly, as shown in Figure 9a, there are two main electrical paths connected in parallel for gas sensing. Path 2 is the normal route along p-type Si, while Path 1 is the one along the discontinuous Pd-decorated SnO_2_ nanofilm electrically connected by conductive p-Si. With respect to Path 2, in an air atmosphere, the oxygen molecules absorbed on the surface would capture electrons from p-type Si along Path 2, thereby increasing the density of holes on the silicon surface and reducing the resistance of Path 2, as these holes serve as charge carriers in p-type semiconductors. However, upon the introduction of H_2_, the trapped electrons would be released back to SiNWs, resulting in a decrease in hole concentration and an increase in the Path 2 resistance [45]. Apparently, Path 2 works the opposite way to the sensors, because experimental results show that the sensor resistance increases in the air atmosphere, while decreasing in the presence of H_2_. Furthermore, bare SiNWs have been prepared for hydrogen sensing, the test results reveal that the response of bare SiNWs is quite low, with a response value of approximately 1.5 towards 1.5% H_2_ at 300 °C (Figure S7), while Sensor 1 (Pd-decorated SnO_2_ based on SiNWs) achieves a response value greater than 9 (Figure 6a). Therefore, it suggests that Path 2 contributes little to the gas sensing. Consequently, Path 1 is likely the dominant electrical path for gas sensing. The hydrogen sensing mechanism can be interpreted according to the working principles of n-type MOS gas sensors [46], which perfectly aligns with what experimental results show. In this study, we focus on the hydrogen sensing mechanism at the temperature of 300 °C, because it is the perfect working temperature for all three kinds of sensors. For pure SnO_2_ nanofilm sensors (Sensor 2 and 3), when they are in an air atmosphere, oxygen molecules are adsorbed onto the oxygen vacancies on the surface of SnO_2_ nanofilm and capture electrons from the SnO_2_ nanofilm, resulting in the formation of O^2−^ and O^−^ ions at 300 °C [47]. This adsorption of oxygen generates an electron-depleted layer at the interface between SnO_2_ nanofilm and oxygen ions, contributing to a reduction in the electron carrier concentration and an increase in the resistance. Subsequently, when hydrogen gas is introduced, it reacts with O^2−^ and O^−^ ions, releasing the trapped electrons back into the SnO_2_ [48], thereby decreasing the thickness of the electron-depleted layer at the interface and the resistance. The relevant reactions are as follows (Equations (1)–(5)):

O_2(gas)_ → 2O_(ads)_ (1)

O_(ads)_ + e^−^ → O^−^_(ads)_ (2)

O_(ads)_ + 2e^−^ → O^2−^_(ads)_ (3)

H_2(gas)_ → H_2(ads)_ (4)

H_2(ads)_ + O^−^_(ads)_ → H_2_O_(gas)_ + e^−^ (5)

According to the measurement results, Sensor 2 with the SiNW structure shows a higher response and baseline resistance than Sensor 3, which could be attributed to the more absorption active sites provided by the larger specific surface area of SiNW structure. Sensor 1 with the decorative Pd particle outperforms Sensor 2 in sensing capabilities including a rapid response time of 9 s, a low detection limit of 1 ppm, and a higher response, which could be caused by three primary factors.

H_2(gas)_ → 2H (6)

2H +O^−^ → H_2_O+ e^−^ (7)

First, the significant improvement in H_2_ sensing behavior could be attributed to the Pd catalytic properties. It promotes the splitting of H_2_ entities into H atoms, which subsequently spread to the SnO_2_ surface through the spillover effect (Equation (6)) [48]. These H atoms react with oxygen ions, releasing free electrons back into the SnO_2_ (Equation (7)). The related sensing mechanism is illustrated in Figure 9b [48]. The fundamental working principles of Sensor 1 are basically similar to those of Sensor 2 and 3. However, the presence of Pd lowers the activation energy needed for these reactions and increases the absorption speed. These may lead to a significantly enhanced response magnitude and the rapid respond for the Pd/SnO_2_ system.

**Figure 10:**
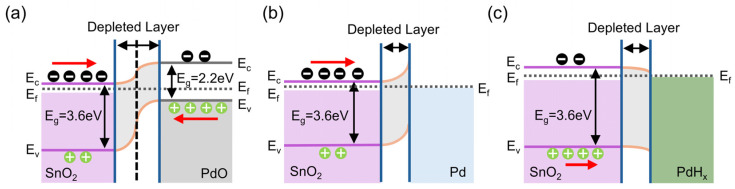
The energy band diagrams of (**a**) SnO_2_/PdO, (**b**) SnO_2_/Pd, and (**c**) SnO_2_/PdH_x_.

Second, the resistance modulations of both the Schottky barrier and p-n heterojunction are crucial to the enhanced gas sensing performance of Sensor 1. XPS analysis indicates that a portion of palladium (Pd) exists as a p-type semiconductor PdO. Furthermore, at 300 °C, in an air atmosphere, a certain amount of PdO is formed through the oxidation of Pd, while some Pd is formed by the reduction in PdO in a hydrogen atmosphere. When PdO contacts the SnO_2_ surface, it acquires electrons from SnO_2_ due to its higher work function compared to that of SnO_2_ [42,49], thereby generating an electron-depleted layer and a p-n heterojunction at the interface between PdO and SnO_2_ (Figure 10a). Meanwhile, the Schottky barrier would be formed at the Pd/SnO_2_ interface as well, because the work function of Pd is higher than that of SnO_2_, and Pd could obtain electrons from SnO_2_ (Figure 10b) [42,49]. Both the p-n heterojunction and Schottky barrier contribute to the formation of electron-depleted layers, resulting in an increase in resistance. Notably, in an air atmosphere, the thickness of these electron-depleted layers is greater than that in a hydrogen atmosphere, leading to higher resistance because of oxygen molecules capturing electrons from the SnO_2_ nanofilm (Figure 9b). This completely explains why the baseline resistance of Sensor 1 is higher than Sensor 2 and Sensor 3. Consequently, both the p-n heterojunction and Schottky barrier enhance the hydrogen sensing response by modulating the resistance of the sensitive materials. Additionally, the hydrogen absorption capacity of Pd nanoparticles further enhances the sensing performance of Sensor 1. Under H_2_ exposure, a certain amount of H atoms generated from the splitting of hydrogen molecules absorbed on the Pd surface would penetrate the lattice of Pd, resulting in the formation of PdH_x_. At the PdH_x_/SnO_2_ interface, SnO_2_ would acquire electrons from PdH_x_ due to its higher work function compared to that of SnO_2_ [49,50], which consequently reduces the thickness of the electron-depleted layer at the PdH_x_/SnO_2_ interface, resulting in a decrease in the resistance (Figure 10c). Therefore, the hydrogen absorption capability of Pd NPs leads to a further decrease in resistance, which improves the overall sensing capabilities of Sensor 1.

Lastly, the p-Si substrate plays a crucial role in modulating the sensor’s resistance, thereby enhancing its hydrogen sensing capabilities. A p-n heterojunction forms at the interface between the SnO_2_ nanofilm (with n-type conductivity) and the p-Si substrate due to their differing work functions. This leads to the formation of an electron depletion layer on the SnO_2_ side and a hole depletion layer on the p-Si side. When exposed to the air, electrons from the SnO_2_ surface are attracted to absorbed oxygen molecules, causing the depletion layer to thicken and increasing the overall resistance. In contrast, when hydrogen gas is introduced, hydrogen molecules react with the absorbed oxygen, allowing electrons to return to the SnO_2_, which results in a decrease in resistance (Figure 9) [51].

## 4. Conclusions

We fabricated SiNW-based Pd-decorated SnO_2_ nanofilms via a simple process, mainly consisting of MaCE and electron beam evaporation. Initially, a 30 nm-thick silver nanofilm was deposited onto a silicon substrate by electron beam evaporation and then annealed to generate a silver holey mask for the following MaCE, enabling the creation of vertical SiNWs. Afterwards, layers of a 40 nm-thick SnO_2_ nanofilm and a 1 nm-thick Pd nanofilm were deposited in sequence onto the SiNW substrate. The experimental results indicated that the hydrogen sensors based on Pd-decorated SnO_2_ nanofilms integrated on SiNWs demonstrated significantly better performance compared to those lacking a SiNW structure or Pd decoration. Particularly, the silicon nanowire-based Pd-decorated SnO_2_ nanofilm hydrogen sensor demonstrated a response value greater than 9, a respond time of 9 s to 1.5% H_2_, and a detection limit of 1 ppm. These performance improvements could be mainly attributed to the large surface area of the SiNW structure, the effective spillover mechanism of the dispersed Pd catalyst, the abundant oxygen vacancies of SnO_2_ nanofilm, and Schottky barrier formed at the heterojunction interface between the Pd nanoparticles and the SnO_2_ nanofilm, as well as the p-n heterojunction provided by p-Si and SnO_2._ This work may establish a foundation for other researchers to investigate future high-performance hydrogens sensors based on the combination of SiNWs, MOS, and noble metals.

## Figures and Tables

**Table 1:** Seven sensors with different thicknesses of SnO_2_ and Pd films.

Sensor	Description
Sensor 1	SiNW-based Pd (1 nm)-decorated SnO_2_ naofilm (40 nm)
Sensor 2	SiNW-based SnO_2_ nanofilm (40 nm)
Sensor 3	40 nm-thick SnO_2_ nanofilm without SiNW substrate and Pd decoration
Sensor 4	SiNW-based SnO_2_ nanofilm (30 nm)
Sensor 5	SiNW-based SnO_2_ nanofilm (50 nm)
Sensor 6	SiNW-based Pd (3 nm)-decorated SnO_2_ nanofilm (40 nm)
Sensor 7	SiNW-based Pd (6 nm)-decorated SnO_2_ nanofilm (40 nm)

## Data Availability

The authors confirm that the data supporting the findings of this study are available within the article.

## Supplementary Materials

The following supporting information can be downloaded at: https://www.mdpi.com/article/10.3390/s25030655/s1, Figure S1: Optoelectrical integrated test platform; Figure S2: SEM images of annealed Ag nanofilms with various thickness; Figure S3: SEM images of 30nm-thick Ag nanofilms annealed at different temperatures; Figure S4: SEM images of 130 nm-thick SnO_2_ deposited on SiNWs; Figure S5: Response and recovery time graphs of Sensor 4 and Sensor 5; Figure S6: Response and recovery time graphs of Sensor 5 and Sensor 6; Figure S7: Response graph of bare SiNWs towards 1.5% H_2_ at 300 °C; Table S1: Detection limits of H_2_ sensors based on SnO_2_. References [21,52,53,54,55] are mentioned in supplementary materials.

## Abbreviations

The following abbreviations are used in this manuscript: SiNWs, silicon nanowires; MaCE, metal-assisted chemical etching; SEM, scanning electron microscopy; XPS, X-ray photoelectron spectroscopy; MOS, metal oxide semiconductor; VLS, vapor–liquid–solid synthesis; RIE, reactive-ion etching; XRD, X-ray diffraction.
