# Supplementary material for: Pd-Decorated SnO2 Nanofilm Integrated on Silicon Nanowires for Enhanced Hydrogen Sensing
Source: Sensors (Basel). 2025 Jan 23;25(3):655. doi: 10.3390/s25030655 (PMC11820637; doi:10.3390/s25030655)
Supplement: Supplementary file 1 [file sensors-25-00655-s001.zip › sensors-3366960-supplementary.pdf]

sensors

MDPI

Supplementary Materials

Pd-decorated SnO_2 Nanofilm Integrated on Silicon Nanowires for Enhanced Hydrogen Sensing

Tiejun Fang[1], Tianyang Mo[1], Xianwu Xu[1], Hongwei Tao[2], Hongbo Wang[2], Bingjun Yu[1], and Zhi-Jun Zhao[1,2,*]

[1] Tribology Research Institute, School of Mechanical Engineering, Southwest Jiaotong University, Chengdu, 610031, China

[2] Institute of Smart City and Intelligent Transportation, Southwest Jiaotong University, Chengdu, 611756, China

* Correspondence: Zhaozhijun@swjtu.edu.cn (Prof. Zhao)

 https://doi.org/10.3390/xxxxx

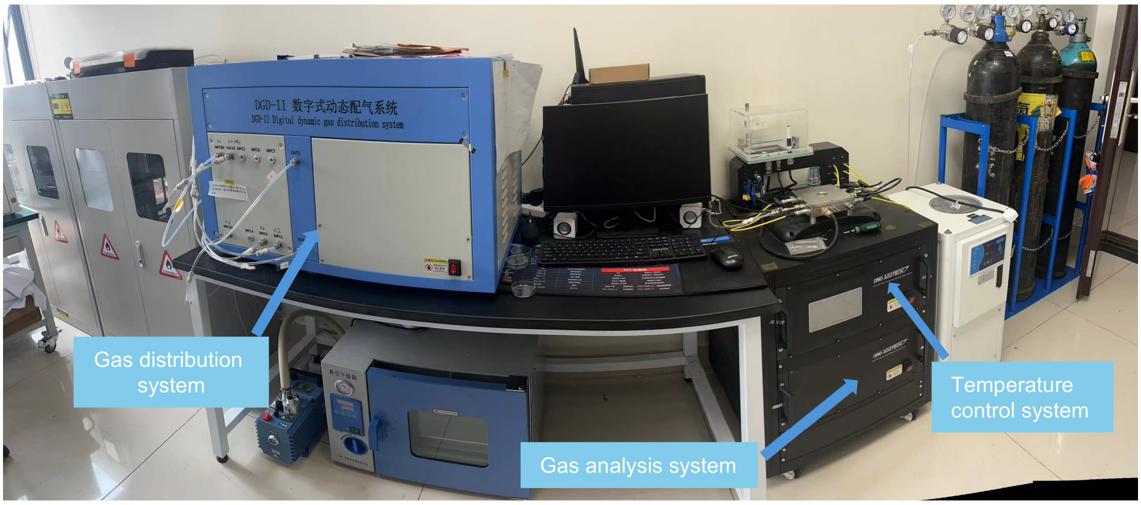

Figure S1. Optoelectrical integrated test platform.

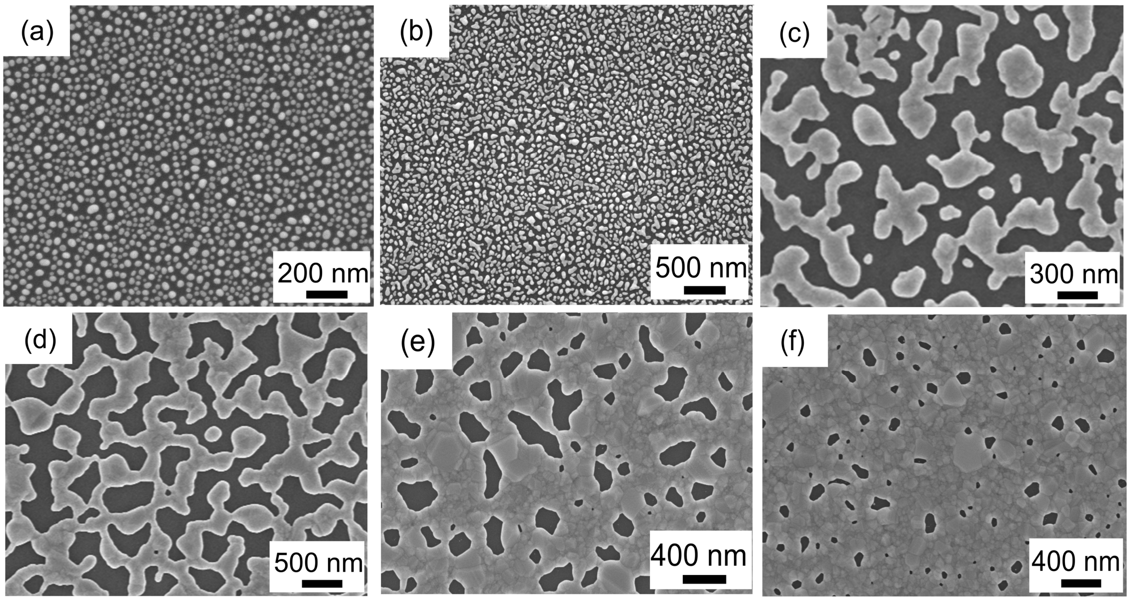

Figure S2. Top-view SEM images of Ag nanofilms with various thicknesses after annealing at 250°C for 30 minutes: (a) 10 nm, (b) 20 nm, (c) 25 nm, (d) 30 nm, (e) 35 nm, and (f) 40 nm.

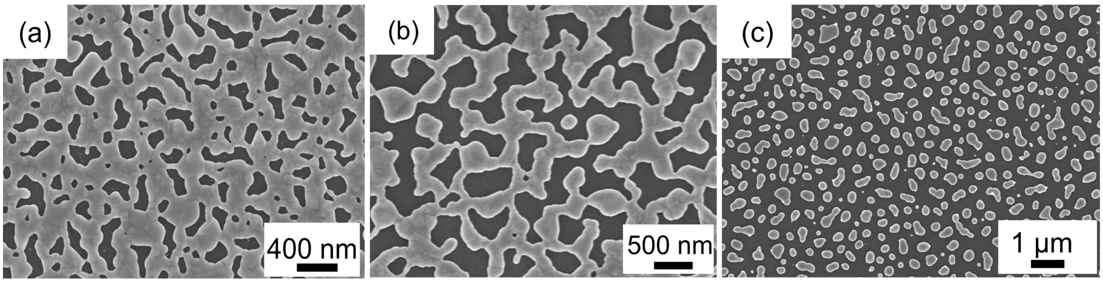

Figure S3. Top-view SEM images of 30 nm-thick Ag nanofilms annealed at 180°C (a), 250°C (b), and 350°C (c) for 30 minutes, respectively.

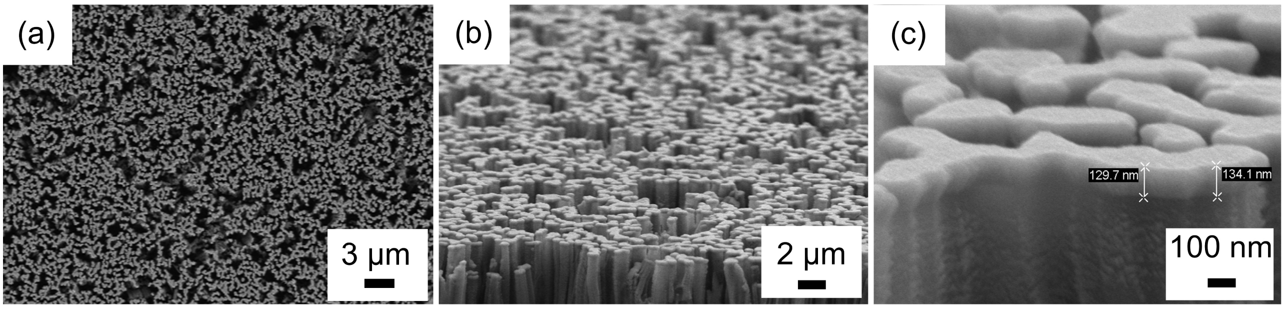

Figure S4. SEM images of 130 nm-thick SnO_2 deposited on SiNWs.

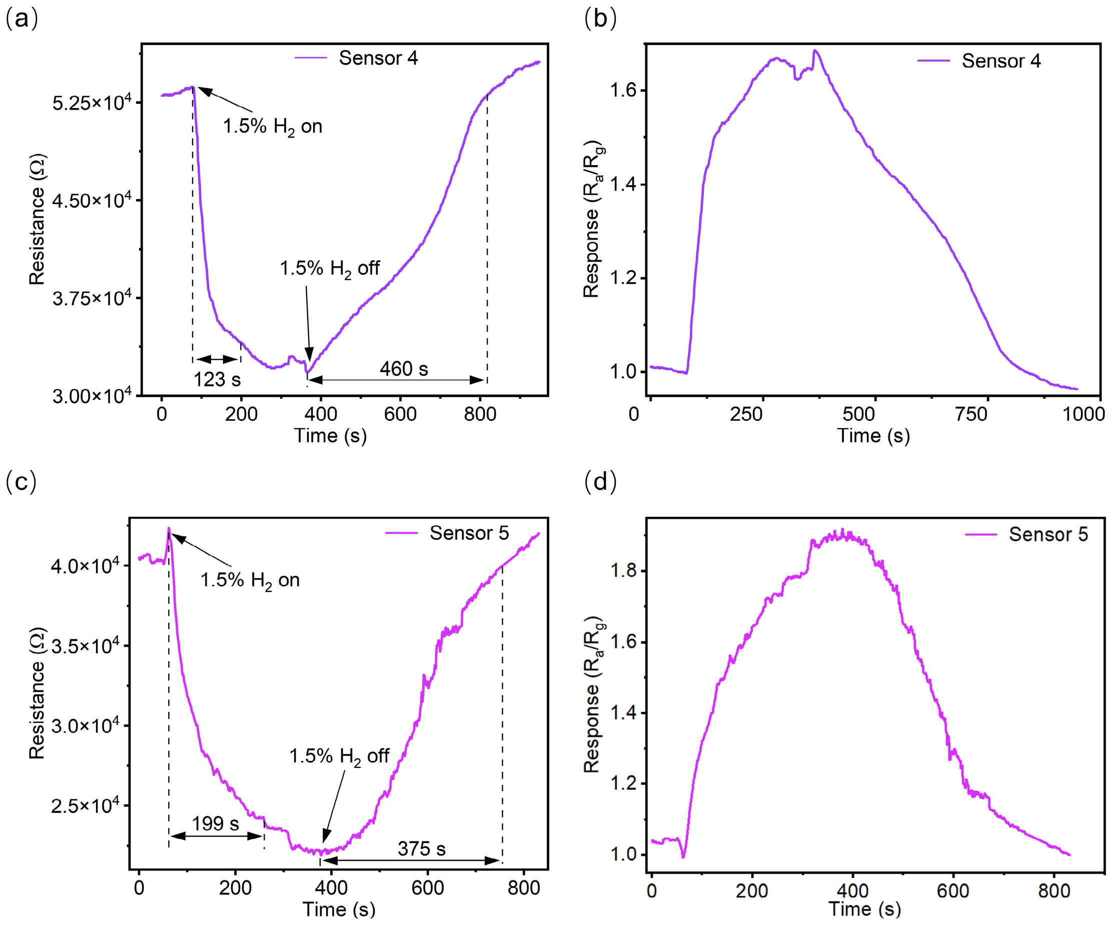

Figure S5. Response and recovery time graph (a), response graph (b) of Sensor 4 towards 1.5% H_2 at 300℃; response and recovery time graph (c), response graph (d) of Sensor 5 towards 1.5% H_2 at 300℃.

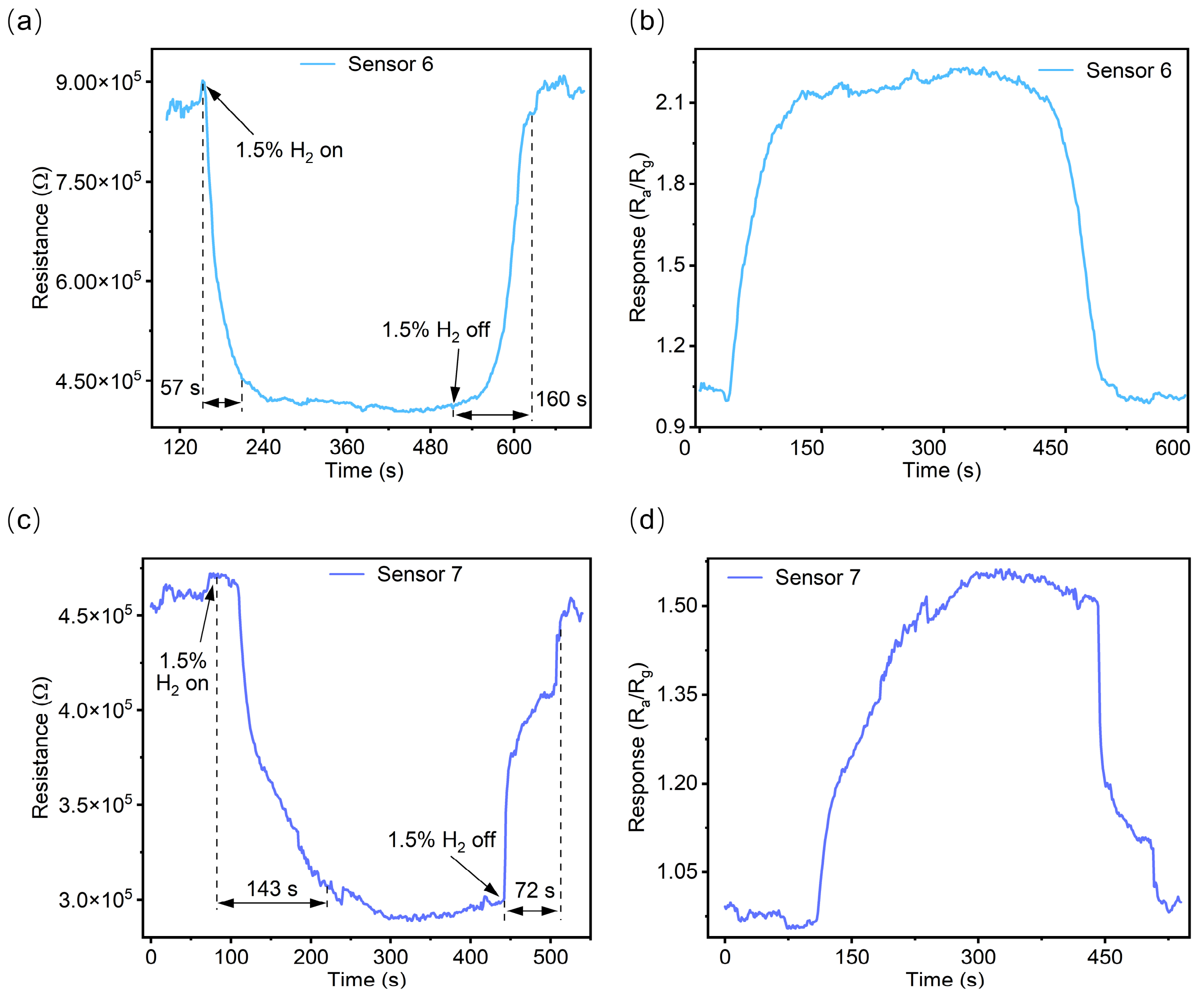

Figure S6. Response and recovery time graph (a), response graph (b) of Sensor 6 toward 1.5% H_2 at 300℃; response and recovery time graph (c), response graph (d) of Sensor 7 1.5% H_2 at 300℃.

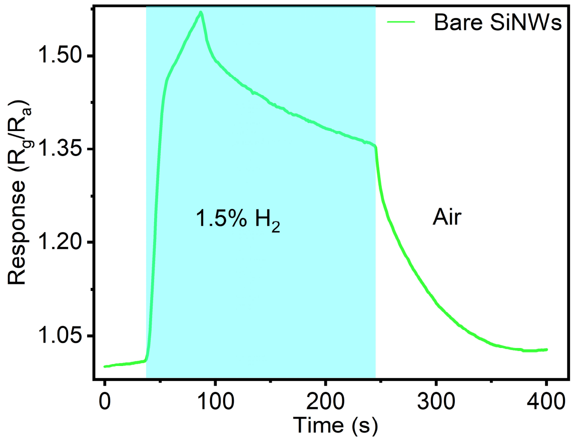

Figure S7. Response graph of bare SiNWs towards 1.5% H_2 at 300℃.

Table S1. Detection limits of H_2 sensors based on SnO_2.

Sensor	Limit of Detection (ppm)	T (°C)	Reference
0.50 at.% Pd/SnO_2	10	125	[1]
Pd/SnO_2 thin film	25	300	[2]
1.0 at. % Pd/SnS_2/SnO_2	10	300	[3]
$Pd_1Ag_{0.50}$@SnO/SnO_2	25	225	[4]
Pd-SnO_2 composite nanofiber	20	280	[5]
SiNW-based Pd-decorated SnO_2 nanofilm	1	300	This work

Reference

1. Meng, X.; Bi, M.; Xiao, Q.; Gao, W. Ultra-Fast Response and Highly Selectivity Hydrogen Gas Sensor Based on Pd/SnO_2 Nanoparticles. *Int. J. Hydrog. Energy* **2022**, *47* (5), 3157–3169.
2. Van Toan N, Chien N V, Van Duy N, et al. Fabrication of highly sensitive and selective H_2 gas sensor based on SnO2 thin film sensitized with microsized Pd islands[J]. Journal of hazardous materials, **2016**, 301: 433-442.
3. Meng X, Bi M, Xiao Q, et al. Ultrasensitive gas sensor based on Pd/SnS_2/SnO_2 nanocomposites for rapid detection of H_2[J]. Sensors and Actuators B: Chemical, **2022**, 359: 131612.
4. Pandey G, Bhardwaj M, Kumar S, et al. Synergistic effects of Pd-Ag decoration on SnO/SnO_2 nanosheets for enhanced hydrogen sensing[J]. Sensors and Actuators B: Chemical, **2024**, 402: 135062.
5. Zhang H, Li Z, Liu L, et al. Enhancement of hydrogen monitoring properties based on Pd–SnO_2 composite nanofibers[J]. Sensors and Actuators B: Chemical, **2010**, 147(1): 111-115.
